# Therapeutic Potential and Main Methods of Obtaining Selenium Nanoparticles

**DOI:** 10.3390/ijms221910808

**Published:** 2021-10-06

**Authors:** Elena G. Varlamova, Egor A. Turovsky, Ekaterina V. Blinova

**Affiliations:** 1 Institute of Cell Biophysics of the Russian Academy of Sciences, Federal Research Center “Pushchino Scientific Center for Biological Research of the Russian Academy of Sciences”, 142290 Pushchino, Russia; 2 Department of Clinical Anatomy and Operative Surgery, Department of Pharmacological Technology and Pharmacology, Sechenov University, 8/1 Trubetzkaya Street, 119991 Moscow, Russia; bevsechenov@msil.ru

**Keywords:** selenium, selenium nanoparticles, therapeutic potential

## Abstract

This review presents the latest data on the importance of selenium nanoparticles in human health, their use in medicine, and the main known methods of their production by various methods. In recent years, a multifaceted study of nanoscale complexes in medicine, including selenium nanoparticles, has become very important in view of a number of positive features that make it possible to create new drugs based on them or significantly improve the properties of existing drugs. It is known that selenium is an essential trace element that is part of key antioxidant enzymes. In mammals, there are 25 selenoproteins, in which selenium is a key component of the active site. The important role of selenium in human health has been repeatedly proven by several hundred works in the past few decades; in recent years, the study of selenium nanocomplexes has become the focus of researchers. A large amount of accumulated data requires generalization and systematization in order to improve understanding of the key mechanisms and prospects for the use of selenium nanoparticles in medicine, which is the purpose of this review.

## 1. Introduction

The trace element selenium (Se) has a pleiotropic effect and has a high therapeutic potential for the treatment of various diseases; therefore, it attracts a lot of attention in biotherapy and nanomedicine. According to the World Health Organization (WHO), the Se consumption rate is 55 µg/day for adult women and 70 µg/day for adult men [1]. According to various sources, the upper permissible level of Se consumption can range from 300 to 600 μg/day [2,3]. The toxic dose is considered to be 900 μg/day. Thus, the borderline between therapeutic and toxic doses is very narrow.

It is believed that the main mechanism of Se cytotoxicity is the generation of oxidative stress due to the intracellular redox cycle of selenide-Se metabolites with oxygen and cellular thiols, which leads to the formation of nonstoichiometric amounts of superoxide and cellular disulfides. The best-studied inorganic Se compounds are Se dioxide and selenites, especially sodium selenite and sodium selenate. It has been shown that Se dioxide can prevent the oxidative damage of DNA caused by the interaction of Fe (II), Cr (III) and Cu (II) with hydrogen peroxide by forming a coordination complex with metals [4,5]. Among all known organic Se-containing compounds, a special place is occupied by methylseleninic acid (MSA), which is formed as a result of the oxidative decomposition of methyl selenocysteine and has a number of important advantages over other selenium agents [6]. 

Selenium nanoparticles (SeNP) have significant advantages over other Se-containing compounds. Firstly, SeNP are low-toxic; even small concentrations can have a cytotoxic effect. Secondly, SeNP have selective cytotoxicity: they lead to the death of cancer cells and do not have a cytotoxic effect on normal cells. The therapeutic potential of SeNP has been actively studied in recent decades. Their important role in the immune system, various neurological diseases, diabetes mellitus, and oncological diseases of various etiologies has been experimentally proven. In addition to this, SeNP have a polyvalent surface, which allows them to interact with various chemical compounds through covalent and non-covalent bonds. The charges on their surface can conjugate with various positively and negatively charged groups (NH, C=O, COO^−^, C–N, etc.), which indicates their high adsorption capacity [7].

Nevertheless, some toxic effects have been identified for SeNP, which may limit their use or require many years of clinical research. The toxic effects of SeNP are primarily associated with their pro-oxidant properties and the ability to disrupt the integrity of the cell membrane [8,9]. It has been shown on *Zebra fish* embryos that 5–10 µg of SeNP does not cause death and developmental disorders, but 20–25 µg of SeNP leads to a number of abnormalities (blood congestion at the cardiac inflow tract and resultant pericardial edema and decrease of heart rate, and tail malfunction) [10]. In *Daphnia magna* and marine bacterium *Vibrio fischeri*, dose-dependent aquatic toxicity of SeNP, including doped nanoparticles, has also been shown [9,11]. In vivo studies in rats have also revealed some cytotoxicity of SeNP at high doses. It was found that the use of high doses of SeNP leads to an excessive accumulation of Se in the kidneys and liver, which can lead to their damage as a result of oxidative stress [12]. In addition, a correlation was shown using high doses of SeNP and animal body weight. While lower intakes of SeNP (0.2 and 0.4 mg Se/kg) resulted in increased body weight, higher intakes (2.0–8.0 mg Se/kg) resulted in decreased body weight, which suggests SeNP toxicity [13]. It was demonstrated that SeNP potential may directly link to human DNA, affecting its structure into a coiled and twisted form, leading to DNA damage; however, this is still a manifestation of high doses [14].

This review presents the latest data on the importance of SeNP in human health, their use in medicine, and the main known methods of their production by various methods.

## 2. The Main Methods of Obtaining Selenium Nanoparticles

**Figure 1:**
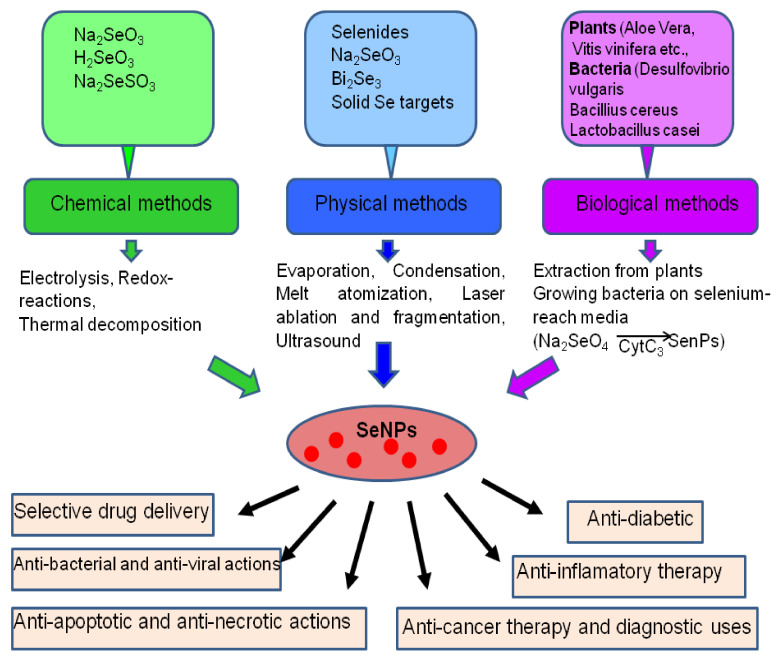
Methods for obtaining SeNPs and their uses.

The main methods for obtaining SeNP are physical, chemical, and biological (Figure 1). In the chemical synthesis of SeNP, sodium selenite, selenous acid, and sodium selenosulfate can be used as the main sources of Se [15,16]. Since it is known that SeNP tend to aggregate in an aqueous medium, they are usually functionalized with suitable stabilizing agents, such as, for example, polysaccharides, quercetin, gallic and ascorbic acids, polyvinyl alcohol, etc. [16,17,18].

Se possesses relatively high photoconductivity, catalytic activity, and oxidative reactions; high piezoelectric, thermoelectric, and nonlinear optical properties; and a low melting point. In addition, the electrical conductivity of Se can be increased by several orders of magnitude when exposed to visible light. All the listed properties of Se make it possible to synthesize nanoparticles on its basis by physical methods. The widespread physical methods for obtaining SeNP are laser ablation and ultrasound.

Laser ablation is a method of removing a substance from a surface by a laser pulse, the cause of which is the reaction of breaking polymer chains inside the irradiated volume due to a reaction activated by laser heating. A possible mechanism for the formation of a colloidal Se in a liquid can be described as follows: a Rayleigh wave of a certain amplitude propagates from the spot of exposure to laser radiation over the surface of the Se. This can lead to ablation of particles of matter from the surface of a solid. As in the case of noble metal ablation, melting and crushing of large selenium particles already exposed to laser radiation in a liquid medium can quickly create high quality nanoparticles without any chemical contamination and in one stage [19,20].

The use of ultrasound in the production of nanomaterials has many beneficial effects. The first direction of application is the use of ultrasound in the synthesis and deposition of nanoparticles. The second is the dispersion of nanoparticles in a liquid to destroy their agglomerates. The impact of ultrasonic radiation is associated, first of all, with the development of such an effect as acoustic cavitation, which occurs in the medium during the propagation of ultrasound [21]. Acoustic cavitation is an effective means of concentrating the energy of a low-density sound wave into a high-energy density associated with pulsations and collapse of cavitation bubbles.

The biosynthesis of nanoparticles using plant extracts and microorganisms has recently become one of the alternatives to chemical and physical methods for obtaining SeNP [22]. One of the advantages of plant-based SeNP biosynthesis is that this strategy is environmentally friendly and economical as it includes natural stabilizers and reductants. Aloe vera leaf extracts have proven themselves as good SeNP reductants and stabilizers as a source of sterols, polysaccharides, vitamins, phenolic compounds, lignin, flavonoids, and proteins [23,24,25]. Also, the biosynthesis of SeNP has been conducted with the use of the extracts of *Vitis vinifera* [26], *Allium sativum* [27], *Dillenia indica* [28], fresh citrus and lemon fruits [29], *Roselle* plant [30], *Cinnamomum zeylanicum* bark, *Prunus amygdalus* leaf [31], and others.

In recent years, SeNP has become more and more widely used in medicine, for cancer therapy, as anti-inflammatory and anti-apoptotic drugs, for the selective delivery of drugs into tissue, for the treatment of diabetes, neurological diseases of the brain, and also as anti-bacterial and antiviral agents (Figure 1).

Characterization and determination of the physical properties of SeNP are an important applied and theoretical problem, as the size and shape of SeNP can determine the physiological effects that that these nanoparticles will have on cells and tissues. Important characteristics for SeNP during their preparation and storage are the concentration, temperature, nature of the biomolecules, and pH of the reaction mixture. For example, spherical SeNP have been proven to have high biological applications, while Se nanowires have high photoconductivity [32,33]. The size of SeNP can determine their antioxidant properties. It has been shown that SeNP scavenge the free radicals in vitro in a size dependent manner at 5 nm–200 nm. Small sized (5–15 nm) SeNP have better free radical scavenging capacity and prevent the oxidation of DNA [34]. The size and shape of SeNP also strongly affects the efficiency of their functionalization with other active substances. Thus, it has been shown that the effectiveness of chitosan as an antitumor and antioxidant compound strongly depends on the characteristics of SeNP [35]. The method of their production also affects the size and shape of SeNP, which can significantly determine their pharmacological properties [36]. Using biosynthesis or chemical synthesis, SeNP with a hexagonal ring structure and rod-like, spherical, or flower-like shapes can be produced. Spherical SeNP have been used for biological and medicinal purposes [37], but there are SeNP with other shapes, such as cubic-like, nanorods, nanowires, nanotubes, nanoribbons, and nanoneedles [33].

## 3. The Role of SeNP in Medicine and Human Health

### 3.1. Role of SeNP in the Immune System

**Figure 2:**
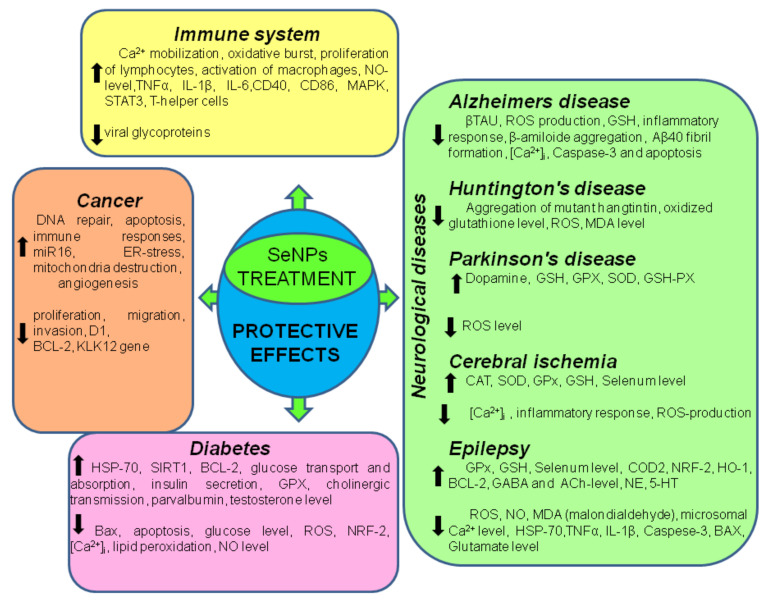
Known beneficial effects of SeNP in various diseases.

It is known that Se plays an important role in inflammation and immunity, participating in the regulation of the immune response and chronic inflammation. At the cellular level, dietary Se can affect various leukocyte effector functions, including migration and cytokine secretion. In addition, there are important links between the functions of selenoproteins and calcium flux, which regulates the oxidative release required for the activation of immune cells. Immune cells require an influx of extracellular calcium to initiate and propagate signals that regulate various functions, including gene transcription, proliferation, chemotaxis, cytokine secretion, and oxidative destruction of phagocytosed microbes (Table 1, Figure 2). Calcium flux is usually generated within a few seconds after stimulation of the receptor in immune cells and is required to generate effective oxidative stress. Thus, increased consumption of Se increases the mobilization of calcium ions, oxidative stress, and translocation of the nuclear factor of activated T cells. In mice, consuming 2 ppm of Se for 8 weeks, or the addition of 100 nM Se ex vivo, resulted in an increase in the expression of the α-subunit of the IL-2 receptor (CD25), as well as in an increase in the proliferative capacity of lymphocytes [38]. T-cells lacking selenoproteins have been shown to exhibit increased levels of oxidative stress and decreased proliferative capacity, suggesting that free thiols are the key mechanism by which dietary Se affects T-cell activation [39].

Macrophages exist in almost all body tissues and play an important role in the innate and adaptive immune response [40]; they are able to synthesize NO, which is involved in the regulation of apoptosis and the protection of cells from microorganisms and tumor cells [41,42]. The activation of macrophages, stimulation of NO release, and increased production of TNF-α, IL 1-β, and IL-6 cytokines is caused by two types of Se: Se-HEP-PLGA (poly (lactic-co-glycolic acid) nanoparticles in which Se-HEP (Hericium erinaceus selenized polysaccharide) is encapsulated and HEP-PLGA-Se (selenium-coated PGLA nanoparticles in which HEP is encapsulated) [43]. However, Se-HEP-PLGA type nanoparticles to a greater extent activate and stimulate macrophages, increasing the expression of CD40 and CD86.

There are many works devoted to the antibacterial action of SeNP, which can damage bacterial cells by various mechanisms and act as an analogue of antibiotics; moreover, SeNP destroy with equal efficiency both gram-positive and gram-negative bacteria strains starting at a concentration of 1 pM [44]. It is known that most bacteria exist in the form of biofilms, specific conglomerates of microorganisms located on some surface, the cells of which are attached to each other, while the cells are usually immersed in the extracellular polymeric substance (extracellular matrix) secreted by them. As a mature biofilm develops, bacteria clump together, forming a barrier that can resist antibiotics and is a source of serious chronic infections. SeNP have shown broad spectrum antibacterial properties against gram-positive and gram-negative bacteria. The mechanisms by which SeNP disrupt bacterial membranes and prevent the formation of biofilms, which significantly reduces the resistance of various bacteria to the action of drugs, firstly, is associated with the activation of ROS production, which causes the damage of cellular components, interruption of transmembrane transport processes, DNA damage, inhibition of enzyme activity, and other effects [45,46,47].

Thus, there was a study of the antimicrobial activity of SeNP against two drug-resistant bacteria, methicillin-resistant *Staphylococcus aureus* (MRSA) and methicillin-resistant *Staphylococcus epidermidis* (MRSE), which cause infections of orthopedic implants, in a model of infected rat femurs. SeNP were shown to slowly release soluble Se species, which inhibited the growth of *Staphylococcus aureus* through mechanisms associated with the depletion of free intracellular thiol [48]. It was shown that SeNP were able to inhibit the formation of biofilms and reduce the number of viable bacteria in the surrounding tissue even at low concentrations of 0.5 ppm. Another study also showed that SeNP, created using a simple colloidal synthesis method, were able to inhibit the growth of *Staphylococcus aureus* and prevent biofilm formation [49].

It is known that the size of nanoparticles is a key parameter that determines their antimicrobial activity: for most nanoparticles, their smaller size correlates with a stronger antimicrobial effect. This statement is also typical for SeNP. Thus, it was shown that nanoparticles with a diameter of 80 nm exhibited the most effective antimicrobial activity [49,50,51,52,53,54]. It was shown that SeNP 43 and 81 nm in diameter increased the production of ROS and changed the potential of the bacterial cell membrane; there was a violation of the metabolism of *Staphylococcus aureus* bacteria due to the depletion of intracellular ATP, which led to a significant decrease in the viability of the bacteria [49]. It is known that the membrane potential has a significant effect on the spatial organization of the cytoskeleton and the division of bacterial cells [55].

**Table 1:** Physiological effects of SeNPs associated with immunity regulation.

	Cellular Effects	Ref.
Immune cells	↑ Ca^2+^-mobilization, ROS, NO-release, IL-2 receptor, lymphocytes proliferation, production of TNFα, IL-1β and IL-6, NKG2D, CD16 ↓ PD-1,	[38,41,48,56]
Antibacterial	↑ ROS, DNA damage, ↓free intracellular thiol, enzyme activity	[45,46,47]
Anti-tuberculosis	↑ PI3K/Akt/mTOR	[57,58]
Anti-autoimmune skin diseases	↑ IL-1β, IL-6, IL-17, IL-22, TGF, MAPK, STAT ↓ formation of plaques, erythema, desquamation, Ki67, PCNA, cyclin-D1, mTOR	[59,60,61,62]
Anti-fibrosis	↑ membrane stabilizing capacity, free radical scavenging activity, antioxidative potential and anti-inflammatory action ↓ TGF-β1, inflammation and alveolitis, infiltration of monocytes, granulocytes, leukocytes	[63,64]

Mannosylated SeNP have been used as a prototype for a new strategy for tuberculosis control [57]. It is known that a hallmark of tuberculosis is the limited delivery of drugs to infected macrophages [58], which strongly express mannose receptors; SeNP can serve as an excellent carrier that delivers drugs precisely to macrophages. It has been shown that these nanoparticles preferentially penetrate macrophages, accumulate in lysosomes, and have the ability of significant antimicrobial and bactericidal destruction of *Mycobacterium tuberculosis*, as well as antimicrobial immunity in host cells, which is accompanied by phagosomal-lysosomal fusion of *Mycobacterium tuberculosis*, the induction of the mitochondria signaling pathway PI3K/Akt/mTOR. This bactericidal strategy, which has a wide range of functions of innate immunity, as well as significantly low cytotoxicity, can potentially serve as an effective treatment for tuberculosis.

In addition, the SeNP in the gel have been used to treat an autoimmune chronic skin disease, psoriasis, characterized by hyperproliferation of keratinocytes [59]. Psoriatic keratinocytes have an increased resistance to apoptosis and a sharp progression of the cell cycle due to the amplification of dendritic cells and T cells of the immune system, which release various pro-inflammatory cytokines and chemokines with the simultaneous activation of growth factors, which are involved in the pathogenesis of psoriasis [60,61,62]. Imiquimod (IMQ) was used to induce psoriasis-like skin inflammation in mice. The daily use of the gel with SeNP at doses of 3 and 10 μg/kg helped to reduce the formation of plaques, erythema, and desquamation. It was shown that the use of SeNP significantly reduced splenomegaly, which indicates a facilitation of the inflammatory response; the levels of IL-1β, IL-6, IL-17, IL-22, TGF, and promoters of growth and proliferation markers such as Ki67, PCNA, cyclin-D1, and mTOR were significantly suppressed. The activation of T–helper cells, in particular Th17, is the main source of IL-22, which mainly targets the activation of mitogen-activated protein kinase (MAPK) and a signaling protein and transcriptional activator (STAT3).

A study of the protective effect of SeNP against bleomycin-induced pulmonary fibrosis in the early and late stages was carried out in [65]. Fibrosis of the lungs is the formation of fibrous (scar) tissue in the lungs, which leads to impaired respiratory function. With fibrosis, the elasticity and extensibility of the lung tissue decreases; it is difficult for oxygen and carbon dioxide to pass through the wall of the alveoli (pulmonary vesicles, in which the inhaled air comes into contact with blood). The application of SeNP in the early stages of pulmonary fibrosis led to a decrease in the level of the cytokine TGF-β1, inflammation, and alveolitis; the application of SeNP in the late stages of pulmonary fibrosis did not lead to significant results. A similar anti-inflammatory effect of SeNP also at an early stage of lung damage and in its various areas caused by bleomycin has been demonstrated by other authors [66,67,68,69]. There was also a decrease in the level of TNF-α and TGF-β; the infiltration of monocytes, granulocytes, and leukocytes was weakened; and inflammation was reduced [63,64]. However, it can still be noted that the anti-inflammatory effect and the ability to reduce anti-inflammatory cytokines by SeNP were observed mainly in the early stages of the disease; as the disease progressed and with the onset of fibrotic reactions, treatment was not effective.

There are a number of studies demonstrating the antiviral action of SeNP. The antiviral action of SeNP against the H1N1 virus has been shown. SeNP functionalized with oseltamivir effectively destroyed the virus at low concentrations through the mechanism of the inhibition of glycoproteins hemagglutinin and neuraminidase, as well as through the suppression of virus-induced oxidative stress of infected cells through a signaling cascade involving p53 and AKT [70]. SeNP functionalized with ribavirin protected the lungs from H1N1 by suppressing the expression of poly-ADP-ribose polymerase (PARP), caspase-8, Bax, p38, and JNKandp53 [71]. The use of SeNP for selective delivery of siRNA targeting the enterovirus 71 VP1 gene (PEI-SeNP @ siRNA-VP1) protected cells from enterovirus 71 [72]. Due to the need to create new effective test systems for detecting COVID-19, nanoparticles are also used in this area [73]. SeNP have higher levels of sensitivity and stability and are cheaper than colloidal gold in qualitative lateral flow immunoassays. More than 10 highly sensitive and rapid tests for detecting COVID-19 infection have already been developed based on SeNP [74]. Thus, the antiviral properties and relatively low cost of production of SeNP suggest their potential for use for the development of antiviral vaccines and drugs with targeted delivery of active components to infected cells with the possibility of avoiding their toxic doses.

### 3.2. Role of SeNP in Neurological Diseases

As an important trace element in animals and humans, Se plays an important role in maintaining normal physiological functions of the brain and has a neuroprotective effect, and some selenoproteins are involved in the regulation of neurodegenerative disorders. It is known that Se metabolism in the brain differs from metabolism in other organs, because it is predominantly preserved in the brain in the presence of selenium deficiency [75,76]. In recent decades, the role of SeNP in neurological diseases has been actively studied (Table 2, Figure 2), since neurons are especially vulnerable to damage caused by oxidative stress for a number of reasons, such as high oxygen consumption (approximately 25% of total body consumption), the presence of a large amount of polyunsaturated fatty acids, and low levels of antioxidant enzymes [77,78,79].

Oxidative stress is one of the main factors in the pathogenesis of many neurological diseases; therefore, natural antioxidants are often used for their treatment, but they have low efficiency [75,76], and thus the use of antioxidants in the form of nanosized particles is becoming increasingly popular. However, it has been shown that nanoparticles are most often oxidizing agents; they can cause damage to neurons and impair the cognitive functions of the brain in Alzheimer’s disease (AD) patients [105,106,107,108,109]. It is known that one of the main effects of Se in AD is the inhibition of amyloid-β (Aβ) aggregation and the ability to cross the blood–brain barrier [110]. It was shown that SeNP modified with sialic acid and coated with peptide B6, as well as SeNP stabilized with epigallocatechin-3-gallate [111] had a similar effect. In the study of Se-containing clioquinol derivatives during the oxidation of Aβ induced by Cu^2+^, a positive effect of Se on the activity of absorption of hydrogen peroxide, the production of intracellular ROS, and aggregation of Aβ was observed [112].

It is known that one of the common causes of neurodegenerative diseases is the accumulation of incorrectly folded proteins in the brain and their aggregation. Thus, in AD, recognition of incorrect folding and aggregation of proteins in the brain Aβ can assume an alternative conformation, leading to its aggregation in the form of amyloid plaques. Studies have shown that extremely high concentrations of metal ions such as Cu^2+^, Zn^2+^, and Fe^2+^ can bind to Aβ and co-localize with amyloid plaques [113]. Therefore, the use of metal chelators for AD treatment, for example, clioquinol (CQ), is of considerable interest, but most chelators can also chelate other metal-containing proteins, which is undesirable and can lead to the disruption of normal physiological functions in the body [114,115]. It has been repeatedly demonstrated that SeNP can bind to Aβ and affect metal ions and modify their surfaces, including ligands, charges, or reactivity [116,117]. Thus, it was shown that l-Cys-modified SeNP (Cys-SeNP) are able to inhibit Zn^2+^-induced formation of Aβ40 fibrils [80].

However, recently, preference has been given to the creation of nanoparticles based on natural products for the treatment of AD [118], for example, resveratrol (Res)-polyphenol, which is present in many plants and drinks and has a wide range of biological effects, the most important of which are antioxidant and neuroprotective properties [119,120]. The creation of SeNP in combination with Res improved the antioxidant and antiaggregatory properties of Res, which was demonstrated on PC12 cells (cell line derived from a pheochromocytoma of the rat adrenal medulla, that have an embryonic origin from the neural crest that has a mixture of neuroblastic cells and eosinophilic cells) [81]. It has been shown that Res@SeNP can bind to the surface of Aβ42 and block the binding of Cu^2+^ to Aβ42 monomers, which damage the cell membrane and lead to cell death. In general, Res@SeNP were found to be more effective than Res alone, which cannot inhibit PC12 cell apoptosis induced by Aβ42-Cu^2+^ aggregates [81].

Chondroitin selenium sulfate (CS@Se) nanoparticles have also been obtained for multipurpose therapy in the treatment of AD [82]. Chondroitin sulfate is a sulfated glycosaminoglycan (GAG) that attaches to the protein core and forms the proteoglycan chondroitin sulfate (CSPG). CSPG is the main component of perineuronal networks [82,121] and is involved in neurogenesis, cell migration, axonal growth, synaptic plasticity, and neuronal regeneration after injuries of the nervous system [122]. Moreover, CS can inhibit the formation of Aβ fibrils [123], block Aβ-induced loss of cell viability and apoptosis, reduce intracellular free calcium concentration and caspase-3 protein expression, and weaken Aβ-induced neurotoxicity both in vitro and in vivo [83]. CS@Se nanoparticles have been successfully synthesized and studied in AD models in vitro [82]. CS@Se has been shown to effectively inhibit the aggregation of amyloid-β (Aβ) and protected SH-SY5Y cells (human neuroblastoma) from Aβ1-42-induced cytotoxicity. In addition, CS@Se reduced the level of ROS and malondialdehyde (MDA) and increased the level of glutathione peroxidase (GSH–Px), as well as decreased the hyperphosphorylation of tau (Ser396/Ser404) [82].

Recent studies have demonstrated a protective role for Se in Huntington’s disease (HD), an inherited neurodegenerative disease that leads to the death of brain cells and is associated with motor, cognitive, and psychiatric disorders in adult patients. This autosomal dominant disease is characterized by variable length CAG trinucleotide repeats, the transcript encoding the huntingtin protein (HTT). Cumulative oxidative stress can damage cellular structures and the DNA repair system, and induce mitochondrial dysfunction, which are considered important factors in neurodegenerative disorders, including HD. It was shown that sodium selenite can reduce the aggregation of mutant huntingtin and reduce the level of oxidized glutathione in the brain of HD mice [124]. There is currently no effective therapy to cure or stop HD progression. The use of SeNP in HD therapy has been proposed [84] for *Caenorhabditis elegans* (*C. elegans*), one of the classical models. The transgenic Huntington *C. elegans* strain HA759 (the *C. elegans* huntingtin fragments were replaced by human fragments and were largely expressed in the ASH neuron) was adapted to study the protection of SeNP against neuronal damage. SeNP at concentrations below 2 μM has been shown to reduce neuronal death, alleviate behavioral dysfunction, and have a protective effect of *C. elegans* under conditions of oxidative stress [84]. The treatment with nanoparticles contributed to a decrease in the ROS level, which indicates their antioxidant function and prevented the aggregation of mutant HTT in vivo.

Parkinson’s disease (PD) is the second progressive neurodegenerative disease after AD disease [125]. Clinically, the main characteristics of PD are dyskinesia with tremors, bradykinesia, muscle rigidity, and postural instability [85,86]. The pathogenesis of PD is still unclear, but various studies have shown that oxidative stress is an important pathological tool of PD, which causes neuronal death and apoptosis [87,88]. MPTP (1-methyl-4-phenyl-1,2,3,6-tetrahydropyridine), a well-known neurotoxin, is a model for PD research. To evaluate the neuroprotective effects of intragastric administration of glycine-SeNP on oxidative stress and behavioral impairment in PD rats, two animal model groups with and without MPTP were studied [89]. It has been shown that MPTP can induce PD by increasing the activity of oxidative stress, leading to the degeneration of dopamine neurons and neurobehavioral disorders. Glycine-SeNP exerted a protective effect on oxidative stress in neurons by increasing SOD and GSH-PX activity and decreasing MDA levels. Thus, glycine-SeNP can be used as a potential therapeutic agent against PD.

Epilepsy is a chronic neurological disease characterized by behavioral, molecular, and neurochemical changes. Between 0.5% and 1% of the world’s population suffers from epilepsy and this neurological disease is characterized by recurrent and unprovoked seizures. The development of seizures can be associated with several factors, including cerebrovascular disorders, trauma, cancer, oxygen deprivation, infections, and genetic disorders of brain development [126]. Modern antiepileptic drugs are associated with numerous side effects such as memory impairment, fatigue, tremors, gastrointestinal symptoms, osteoporosis, depression, dizziness, and nausea [127]. Due to the high ability of SeNP to cross the blood–brain barrier and their low side effects, they can be regarded as a promising treatment for epilepsy. Neuronal hyperexcitability and epileptogenesis are associated with oxidative stress resulting from mitochondrial and endoplasmic reticulum dysfunction, resulting in the excess production of free radicals that deplete neuronal antioxidant molecules [128,129]. SeNP protect neurons from oxidative damage in epilepsy by inhibiting the byproduct of lipid peroxidation (MDA) and decreasing Hsp70 and NO production, accompanied by increased GSH levels. Se is included in the structure of selenoproteins and selenoenzymes, which are capable of suppressing ROS and, therefore, suppressing the development of oxidative damage [90]. SeNP administration suppressed inflammation in the hippocampal tissue in epilepsy by inhibiting the expression of NF-κB [91,92]. In addition, SeNP contribute to the restoration of the levels of neurotransmitters ACh, NE, DA, 5-HT, and GABA in brain tissue after induced epilepsy, which contributes to the restoration of neuronal connections and the suppression of apoptosis [92,93].

Ischemic cerebral stroke, resulting from various disorders of blood flow, affects millions of people a year around the world. Cerebral ischemia is accompanied by tissue hypoxia and glutamate excitotoxicity, which contribute to the death of brain cells and aggravate the course of strokes. At the same time, the methods and approaches for the treatment and relief of stroke symptoms are significantly limited due to the rapid metabolism in the brain and poor transport of most neuroprotective agents across the blood–brain barrier [92]. Selenium compounds not only suppress the formation of ROS during ischemia/reoxygenation and hypoxia, but also activate mitochondrial biogenesis and, as a consequence, the level of intracellular ATP and Ca^2+^ homeostasis and promote cell survival in the penumbra zone [94,95]. SeNP are able to easily penetrate the blood–brain barrier, while they have powerful antioxidant properties against a background of reduced cytotoxicity compared to selenium compounds, which can help protect brain cells from ischemic damage [96,97]. Of particular interest is the enhancement of BDNF expression in brain cells after exposure to SeNP, which contributes to the suppression of oxidative stress, apoptosis, and inflammation, as well as a decrease in [Ca^2+^]i under the toxic effect of glutamate [98,99,100]. ER-stress occurs during ischemia/reoxygenation, which leads to incorrect folding of proteins in brain cells, impaired Ca^2+^ homeostasis, and the activation of death processes. The ER-stress mechanism involves a number of ER-resident selenoproteins: SELENOK, SELENOS, SELENOM, SELENOT, SELENOF, SELENON, and DIO2, the expression level of which is regulated by the addition of SeNP [101,102,103,104].

### 3.3. The Role of SeNP in Oncology

At present, there is no doubt that Se has anticancer properties, as has been demonstrated by a large number of studies [130]; however, data on the antitumor properties of this microelement are still contradictory and it is difficult to identify the main results. Many studies demonstrate the anticarcinogenic activity of Se associated with the regulation of the expression of redox proteins and modulation of the intracellular redox status [131,132,133,134,135,136,137,138]. In addition, various other mechanisms are involved in the anticarcinogenic effect of Se: the ability to counteract the toxicity of heavy metals, maintain DNA stability, stimulate DNA repair, regulate inflammatory and immune responses, induce cell cycle arrest and apoptosis, block angiogenesis, etc. [130,139,140,141]. One of the reasons for the toxicity of Se for cancer cells as compared to normal somatic cells is the more reducing environment of cancer cells, which stimulates the formation of Se–S adducts from Se compounds [142]. Such Se–S adducts can mimic cystine and mixed disulfides, causing enhanced receptor-mediated uptake of Se by cancer cells [142,143,144,145,146].

SeNP are widely used in medicine, primarily due to the minimal risk compared to Se itself (Table 3, Figure 2). Very often, SeNP are used in combination therapy, which increases the effectiveness of treatment. SeNP are often used as carriers of chemotherapeutic agents such as cisplatin, 5-fluorouracil, doxorubicin, and irinotecan, which demonstrates the presence of a synergistic effect between anticancer drugs and Se, which selectively induces apoptosis [147,148,149,150]. When studying the effect of SeNP in combination with radiation therapy on the example of non-small cell lung cancer, one of the most common cancers in the world, a decrease in cell proliferation, migration, invasion, and apoptosis was shown [151]. In addition, SeNP in combination with radiation therapy contributed to the suppression of the CCND1-important cell cycle protein expression, affecting the cell transition from the G1 phase to the S phase [152], as well as the c-Myc nuclear protein with multiple biological functions. In addition, the zinc-dependent metalloproteinases MMP2 and MMP9 are known to be actively expressed in lung cancer and have also been shown to be inhibited by this combination therapy [147].

It has been repeatedly demonstrated that SeNP exhibit a cytotoxic effect on various prostate cancer cells: PC-3 line [157] and LNCaP [158]. It is known that androgen-dependent prostate cancer can transform into androgen-independent cancer, which does not respond to effective treatment; therefore, there is an urgent need to create effective drugs. It was shown that SeNP had a significant inhibitory effect on prostate cancer cells of the CaP line, regardless of whether they were androgen-dependent, while there was a slight cytotoxicity in normal prostate cells [59,159]. In this study, the association of SeNP with miR-16 miRNA activation was shown, which is closely related to tumor development and to the regulation of the cell cycle and apoptosis [153,154]. It was found that SeNP activate miR16 by suppressing its two key targets: cyclin-D1 and Bcl-2. A positive correlation was found between serum selenium levels and miR16 expression.

Chemotherapy treatment of patients with bone tumors or bone metastases often leads to serious side effects, which include high drug toxicity, induced drug resistance, etc. A new strategy for the treatment of early bone metastases involves the joint targeted delivery of several chemotherapeutic agents. In particular, it was shown that selenite-ligated hydroxyapatite nanoparticles loaded into a hydroxyapatite-binding antitumor platinum complex (PtPP–HASe) selectively reduced the proliferation of cancer cells without reducing the proliferation of bone marrow stem cells [160]. This study was carried out on prostate cancer cells (PC3 cells) and breast cancer cells (MDA-MB-231 cells), culturing them together with bone marrow stem cells (hBMSc cells). The resulting nanoparticles showed a sharp release of Se (within 1 h) and a sustained release of Pt, and selective in vitro cytotoxicity against cancer cells, but not healthy bone marrow stem cells.

A number of works are devoted to the study of the cytotoxic effect of SeNP conjugated with an RGD peptide (RGDfC-SeNP), in which siRNAs are tightly packed to various target genes, using the example of various types of cancer cells [155,161,162]. It is known that RNA interference is one of the promising methods for the treatment of malignant tumors; however, siRNAs in a physiological environment are extremely unstable and susceptible to the action of nucleases; therefore, their delivery to cells using nanoparticles protects them from degradation. The RGD peptide provides binding to integrin αvβ3, which is overexpressed in cancer cells and provides electrostatic interaction of nanoparticles with negatively charged siRNAs due to their positive charge. With the use of three inhibitors of endocytosis, amyloid (macropinocytosis), nystatin (caveola endocytosis) and chlorpromazine (clathrin-associated endocytosis), these functionalized nanoparticles have been shown to enter cancer cells by clathrin-mediated endocytosis. Thus, interfering RNAs selectively inhibiting the KLK12 kallikrein-related peptidases 12-serine protease associated with kallikrein, which is activated in colorectal cancer [163], were selectively delivered to HT-29 cells in cells. Under acidic conditions, which is desirable for the treatment of colorectal cancer, they are rapidly released. These nanoparticles were selectively absorbed by PE-29 cells selectively and did not penetrate into human umbilical vein endothelial cells (HUVEC). In the cytoplasm, siRNA significantly suppressed the KLK12 gene, which contributed to a decrease in proliferation, migration, and invasion in HT-29 cells through the mechanism of mitochondrial destruction mediated by ROS growth [155]. Similar mechanisms of the cytotoxic action of nanoparticles have been described for liver cancer [162], cervical cancer [162], ovarian cancer [164], and small cell lung cancer (NSCLC) [165].

Also, to increase the efficiency and selectivity of siRNA delivery, the synthesis of new layer-by-layer Se-based nanocomplexes (LBL-Se-NC), which consist of three layers [166], was applied. The nucleus is represented by selenium coated with chitosan (Se @ CS), the second layer is siRNA (Se@CS:siRNA), and the third protective layer is chitosan, due to which cellular uptake and endosomal release (Se@CS:siRNA:CS) was carried out. To determine the selective anticancer potential of LBL-Se-NC, the siRNA-chitosan complex (CS-NC) obtained by ion gelation was chosen as a control. It was found that LBL-Se-NC is able to selectively induce apoptosis in 32% of human non-small cell lung carcinoma (H1299 cells), which is 5.7 times more than in normal NIH3T3 cells (mouse embryonic fibroblasts). When cancer cells were treated with the CS-NC complex in the same concentration range, there was no clear difference in the viability of these cells compared to normal cells, which indicated the selective and effective induction of apoptosis by LBL-Se-NC nanoparticles [146,166].

#### Molecular Mechanisms of Anticancer Effects of SeNPs

**Figure 3:**
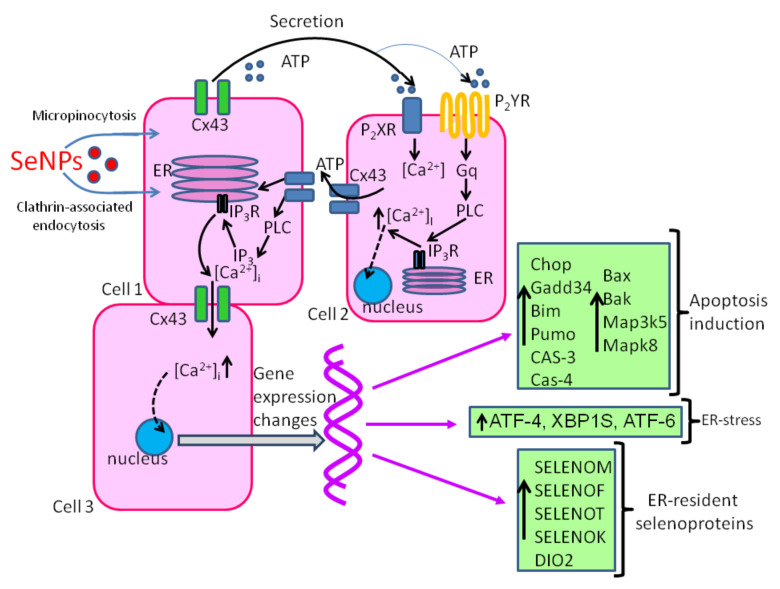
Ca2^+^-dependent mechanism of anticancer action of SeNP. Designations: ER, endoplasmic reticulum; Cx43, connexin hemichannels; IP_3_R-inositol trisphosphate receptor; IP_3_, inositol trisphosphate; RyR, ryanodine receptor, PLC, phospholipase C; ATP, adenosine triphosphate.

Impaired Ca^2+^ homeostasis activates either programmed cell death (apoptosis) or the collapse of the mechanisms for maintaining the physiological functions of the cell (necrosis) [167]. SeNP may be involved in the restoration of calcium homeostasis. SeNP have been shown to enhance the expression of parvalbumin in the brain and have a neuroprotective effect through maintaining Ca^2+^ homeostasis [168], which is a neuroprotective effect and contributes to the suppression of apoptosis and tissue necrosis. However, at the same time, the cytotoxic effect of SeNP is of interest for anticancer therapy. There are three main pathways for the induction of apoptosis: the mitochondrial pathway, the death receptor pathway, and the ER pathway, which ultimately lead to the activation of caspase-3 and proteolysis of cellular components [169]. All these processes are calcium-dependent to one degree or another. In response to the application of SeNP to cells of various cancer lines, a dose-dependent increase in [Ca^2+^]_i_ occurs [101]. The mechanism of increasing Ca^2+^ ions in the cytosol involves the activation of IP_3_ receptors, mobilization of Ca^2+^ from the ER, and activation of intercellular communication through connexin channels and paracrine ATP secretion (Figure 3). This increase in [Ca^2+^]_i_ leads to a change in the expression profile of gene encoding proteins responsible for the induction of apoptosis [156]. Increased expression of the pro-apoptotic genes CHOP, GADD34 BIM, PUMA, and BAX indicates the activation of apoptosis through the mitochondrial pathway. An increase in the expression level of caspase-4 may indicate the activation of the internal ER-mediated apoptosis pathway [170]. At the same time, upregulation of mRNA expression of mitogen-activated kinases in MAP3K5 and MAPK-8 cells suggests the activation of an alternative mitogen-activated kinase pathway for apoptosis [171]. Thus, the anticancer effect of SeNP occurs due to the simultaneous activation of several signaling pathways of apoptosis.

### 3.4. SeNP and Diabetes Mellitus

Diabetes mellitus is a group of endocrine diseases associated with impaired glucose uptake that develops due to insufficiency of the hormone insulin, which is accompanied by hyperglycemia. At the same time, the most common metabolic immune disease worldwide is insulin-dependent type 1 diabetes mellitus, which is characterized by the destruction of pancreatic beta cells due to oxidative stress and apoptosis [172]. The most promising method of treating patients with this disease is currently islet cell transplantation; however, a significant limitation of this approach is the high sensitivity of these cells to various types of stress, which leads to their apoptosis [173].

In addition, it is known that type 1 diabetes develops diabetic nephropathy, which is characterized by the development of sclerosis of the renal glomeruli (glomerulosclerosis), leading to impaired renal function (primarily filtration) and the development of chronic renal failure [174]. In addition, the cause of the pathogenesis of diabetic nephropathy is oxidative stress, which is accompanied by an increase in the glucose content in the kidneys, changes in cellular functions at the molecular level [175,176], and an increased activity of NADPH oxidase, leading to the accumulation of fibronectin and collagen and, as a result, to interstitial fibrosis of the tubules [173]. To date, there is practically no information on the role of SeNP in slowing the progression of diabetic nephropathy in type 1 diabetes mellitus. Based on the antioxidant action of Se compounds and their interaction with heat shock proteins, it has been suggested that SeNP may be effective in the treatment of type 1 diabetic nephropathy caused by streptozotocin (ST3). It was found that in ST3-induced diabetic rats, SeNP significantly influenced the expression of HSP70 (heat shock protein) and SIRT 1 (longevity protein). In addition, there was a decrease in apoptosis in the diabetic kidney due to changes in the expression of Bcl-2 and Bax [177].

Recently, SeNP, a kind of red elemental Se (Se0) in the colloidal state, have attracted more and more attention due to their high bioavailability, biological activity, and low toxicity. The range of acute toxicity of SeNP is 7 times lower than for sodium selenite and 3 times lower than for organic selenium, which was shown in mice (LD50 113, 15, 30–40 mg Se/kg body weight, respectively) [178]. To assess the antidiabetic activity, SeNP stabilized with a polysaccharide from *Catathelasma ventricosum* (CVPs), an edible fungus with powerful antioxidant activity and a protective effect of liver, kidney, and pancreas tissues, were prepared [179]. In addition, such CVP-SeNP have been compared with the antidiabetic activity of other Se preparations, naked SeNP, selenocysteine, sodium selenite, and CVP-SeNP in combination with vitamin E (VE) [180]. It was found that the introduction of CVP-SeNP significantly improved body weight and blood sugar levels and increased the activity of antioxidant enzymes in mice with ST3-induced diabetes, which indicates a pronounced antidiabetic activity of CVPs-SeNP. In addition, the combination of CVPs-SeNP and VE had a stronger effect on the values of these antidiabetic parameters, which indicates their synergism in antidiabetic activity. However, the mechanisms are not well understood. The resulting products were very stable, safe, and could be used as medicines and food in the future [180].

It is known that type 2 diabetes mellitus (non-insulin dependent diabetes) is a metabolic disease characterized by chronic hyperglycemia, which develops as a result of a violation of the interaction of insulin with tissue cells. It has been repeatedly shown that Se can lower glucose levels and has insulin-like activity [181,182]. In addition, selenate has been shown to enhance glucose transport and uptake in rat adipocytes by translocating glucose transporters such as GLUT-1 and GLUT-2 [183]. To evaluate a new therapeutic approach for type 2 diabetes, SeNP were synthesized using glucose as a reducing agent and polyvinyl pyrrolidone (PVP) as a stabilizer. It is known that hyperglycemia and ST3-induced diabetes increase free radicals, which react with polyunsaturated fatty acids in cell membranes, which leads to lipid peroxidation [184]. In this study, SeNP increased insulin secretion and regenerated pancreatic cells. Administration of nanoparticles to animals increased the activity of glutathione peroxidases and reduced oxidative stress [185].

Chitosan-stabilized SeNP (CTS-SeNP) has been shown to increase the antidiabetic effect of metoformin (MT), a sugar-lowering drug of the biguanide class used in the treatment of type 2 diabetes mellitus, especially in overweight and obese individuals, and at the same time preserved normal renal function [186]. Combined therapy with CTS-SeNP and MT contributed to a decrease in heart and renal failure, lipid accumulation, and the level of pro-inflammatory cytokines caused by an increase in glucose levels and the restoration of antioxidant capacity. The causes of liver damage caused by diabetes 2 are mainly related to insulin resistance and subsequent hyperglycemia, lipid and carbohydrate metabolism disorders, which lead to excessive oxidative stress and inflammation of the liver tissue. Treatment of ST3-induced rats with mono- or combination therapy (MET and/or CTS-SeNP) resulted in normal levels of diabetes biomarker activity: AST (aspartate transaminase), ALT (alanine transaminase), GGT (gamma glutamyl transpeptidase), and ALP (alkaline phosphatase), which are closely associated with liver tissue damage [186].

Although the brain requires a great deal of glucose to function properly, neurotoxicity is observed in hyperglycemia caused by the constant consumption of glucose in a diabetic cell. There are a number of parameters for understanding and assessing the severity of diabetes, such as NFBG (non-fasting blood glucose) and FBG (fasting blood glucose). Treatment of ST3-induced rats with mono- or combination therapy (MET and/or SeNP) was accompanied by a decrease in blood glucose levels starting from the second week and throughout the entire intervention [187]. There was also an increase in body weight and normalization of glucose levels, which may indicate an improvement in metabolic status and a decrease in the tissue damage associated with hyperglycemia. Maintaining glucose homeostasis is essential for reducing the risk of microvascular or macrovascular complications in diabetes [188]. Thus, in the groups of animals treated with SeNP and/or MT, there was an increase in insulin concentration and a decrease in the HOMA index (insulin resistance index) [187]. Brain cell membranes are rich in peroxide fatty acids, which undergo peroxidation under oxidative stress [189]. Treatment of rats with ST3-induced diabetes led to a decrease in lipid peroxidation due to a decrease in MDA (malondialdehyde) levels, a decrease in myeloperoxidase and acetylcholinesterase activities in brain samples, which indicates the induction of an inflammatory response in diabetic neurotoxicity, an improvement in cholinergic neurotransmission, and the restoration of support-motor functions [187]. In addition, SeNP and/or MT therapy led to a significant decrease in the expression of NF-E2-related factor 2 (Nrf2), an essential transcription factor that regulates an array of detoxifying and antioxidant defense gene expression in the liver. It is activated in response to oxidative stress and induces the expression of its target genes by binding to the antioxidant response element (ARE) [187]. This may serve as one of the mechanisms or pathways involved in ameliorating diabetes-mediated neurobehavioral dysfunction. Decreased levels of the calcium-binding protein parvalbumin in brain samples are also known to be associated with diabetes [190]. The observed increase in parvalbumin expression after treatment with SeNP and/or M confirms their beneficial neurotherapeutic effects, accompanied by modulation of calcium homeostasis, which represents another possible pathway for the regulation of brain damage by SeNP caused by diabetes mellitus [187].

Also, stable nanoparticles functionalized with polysaccharide from the fruit of R. roxburghii (RTFP–3) had good antioxidant properties and inhibited α-glucosidase, H_2_O_2_-induced apoptosis of INS-1 cells by suppressing oxidative stress and expression of UCP-2 protein (mitochondrial uncoupling protein 2), which belongs to the family of mitochondrial anion carrier proteins [191]. The main function of proteins of this family is the separation of oxidative phosphorylation and ATP synthesis, facilitating the transfer of anions from the inner to the outer mitochondrial membrane and the reverse transfer of protons. A decrease in membrane mitochondria leads, in turn, to a decrease in the production of ROS. Thus, RP3-SeNP may function as a promising candidate for the treatment of ROS-mediated diabetes.

The leaves of Hibiscus sabdariff (roselle plant) were also used to restore SeNP, which is the best crop for developing countries because it is relatively easy to grow. Rosella leaves are mainly composed of ascorbic acid, proteins, and carbohydrates, which play a key role in the synthesis and formation of SeNP [192]. These nanoparticles were studied in diabetic rats with testicular dysfunction. Biochemical studies have shown that such nanoparticles were able to increase serum testosterone levels and significantly reduce testicular oxidative stress, namely nitric oxide and lipid peroxidation. In addition, microscopic studies have shown that SeNP is able to prevent histological damage that occurs in the testes of rats with STZ3-induced diabetes [193].

### 3.5. Role of SeNP in Oxidative Stress, Bone Health, and Inflammation

SeNP, depending on the dose, possess both prooxidant and antioxidant properties. SeNP at a concentration of 12 μM have been shown to promote the antioxidant capacity of cells, while a treatment of 24 μM SeNP damaged the antioxidant capacity of cells [103,194]. In cancer cells, due to the acidic pH state with redox imbalance, SeNP cause ROS overproduction, which leads to disruption of the integrity of mitochondria and ER-stress. This causes cellular stress due to the activation of multiple molecular pathways that include NFκB, MAPK/Erk, Wnt/β-catenin, PI3K/Akt/mTOR, and apoptotic pathways [195]. 

The antioxidant action of SeNP is realized through selenoproteins and a number of enzymes–glutathione peroxidase, thioredoxin reductase and iodothyronine deiodinases. SeNP scavenge wide range ROS, superoxide anion, 1,1-diphenyl-2-picrylhydrazyl, singlet oxygen, carbon-centered free radicals [196,197,198].

If in cancer the activation of oxidative stress is a key link in the therapy with SeNP, in the case of rheumatoid arthritis (RA), on the contrary, suppression of oxidative stress and inflammation is a protective strategy. RA is a common chronic inflammation-mediated and systematic autoimmune disorder. It is a long-lasting condition described as inflammation of diarthrodial joints leading to symmetrical polyarthritis and synovial hyperplasia (swelling) that results in the progressive destruction of cartilage and bones and the loss of articular function that leads to the eventual deformation of joints [36]. SeNP reverted the GPx1, CAT, and COX-2 mRNA expression and restored the levels of TNF-α, IL-1β, IL-6, and MCP-1 [199] and can be a promising method of RA therapy.

SeNP loaded with CAT and functionalized with FA and HA are shown to target activated macrophages associated with rheumatoid arthritis and atherosclerosis via CD44 and FR-βreceptors which are more expressed in these cells. SeNP specifically destroyed pro-inflammatory-activated macrophages responsible for producing high levels of H_2_O_2_, without affecting non-activated macrophages [200].

### 3.6. Future Perspectives

Nanotechnology is widely used for applied problems of modern medicine in order to create effective nanoparticles and nanorobots for the treatment of the body at the cellular level. The final stages of clinical trials are already underway and products using nanoparticles for the early detection of cancer, such as NanoFlares [201], are close to being introduced, which bind to targets on cancer cells and begin to generate light. One of the promising technologies is the use of magnetic nanoparticles and NMR, where nanoparticles attach to the microvesicles of cancer cells in the bloodstream and are easily detected using NMR [202]. Gold nanoparticles developed by Nanosphere have already received results from clinical trials in detecting early stages of cancer. In addition, cadmium-based Qdots (Invitrogen) are already close to being used for diagnostic purposes in humans, but currently only commercial use is allowed for animal experiments. Work is underway to create Qdots based on silicon, which is considered less toxic. In this vein, selenium, which has low cytotoxicity, can activate protective signaling pathways, and is capable of being metabolized and easily excreted from the body, is a prospect.

However, the development of nanomaterials based on the above substances has been going on for a long time in comparison with Se compounds. The creation of Se-based nanoparticles, as well as SeNP doped with active compounds, has provided reliable and convincing results of preclinical studies only in the last few years. Patents have appeared not only for methods of obtaining SeNP, but primarily in two categories: nanoparticle drug delivery and cancer-targeting nanoparticles (US8445026B2, etc.). [203]. The combination of this SeNP property with well-known chemotherapeutic agents, 5-Fluorouracil, doxorubicin, and irinotecan, or with small interfering RNA (siRNA), exhibits synergistic antitumor activity and overcomes multidrug resistance [97,204,205,206,207]. In view of the growing number of studies on the mechanisms of action of doped SeNP, as well as with the development of technologies for creating multilayer SeNP, one should expect the appearance of patents on the creation of effective neuroprotectors and antiepileptic drugs based on SeNP [208]. A limitation for more rapid introduction of SeNP for widespread use in clinics is the need to study their action mechanisms on cells and tissues, especially their toxic properties, and to determine the role of their functionalization by other active compounds on these toxic properties. It is also important to determine the therapeutic window for the effective use of drugs based on SeNP in comparison with conventional therapies. 

In addition to being used in biomedicine, SeNP will find application in the future as a fertilizer and for saturation of plants, as this microelement is necessary for mammals. It was shown that SeNP are able to improve the morphological properties of cultivated plants. The plants’ growth and habitat were slightly better with a SeNP dose of 10 μg/kg. The plant leaf plate surface area after SeNP application was almost 2 times larger compared to the stressed plants grown without SeNP addition to the soil. Thus, SeNP are able to provide long-term fertility of the soil system and high stable productivity of plants [209]. 

## 4. Discussion

Currently, the literature widely discusses the prospects for the use of nanoparticles in the creation of drugs. Selenium is one of the most important trace elements necessary for the normal functioning of human organs. This element plays an important role in metabolism, thyroid function, and protecting cells from damage associated with oxidative stress. It is included in selenoproteins in the form of selenocysteine, which is the most important part of the active center of their enzymatic activity. Many selenoproteins have oxidoreductase activity and thus regulate physiological redox balance. According to various sources, the upper permissible level of Se consumption can range from 300 to 600 μg/day [2,3]. The toxic dose is considered to be 900 µg/day. Thus, the line between therapeutic and toxic doses is very narrow, while SeNP have a markedly reduced toxicity.

This review discusses in detail the main modern methods of obtaining SeNP and their important therapeutic effect in various human diseases. Thus, the formation of SeNP can occur in the course of such processes as phase transformations, chemical interaction, recrystallization, amorphization, high mechanical loads, and biological synthesis. Methods for obtaining SeNP are divided into mechanical, physical, chemical, and biological, i.e., they are based on the nature of the nanoparticle synthesis process. The widespread physical methods for obtaining SeNP are laser ablation and ultrasound. Laser ablation is a method of removing a substance from a surface by a laser pulse, the cause of which is the reaction of breaking polymer chains inside the irradiated volume due to a reaction activated by laser heating. The impact of ultrasonic radiation is associated, first of all, with the development of such an effect as acoustic cavitation, which occurs in the medium during the propagation of ultrasound [21]. Acoustic cavitation is an effective means of concentrating the energy of a low-density sound wave into a high-energy density associated with pulsations and collapse of cavitation bubbles. Biosynthesis of nanoparticles using plant extracts and microorganisms has recently become one of the alternatives to chemical and physical methods for obtaining SeNP [22].

In recent years, SeNP has become more and more widely used in medicine, for cancer therapy, as anti-inflammatory and anti-apoptotic drugs, for the selective delivery of drugs into tissue, for the treatment of diabetes, neurological diseases of the brain, and as anti-bacterial and antiviral agents. The immune-stimulating effect of SeNP have been repeatedly confirmed, and they have demonstrated broad-spectrum antibacterial properties against gram-positive and gram-negative bacteria. In addition, SeNP in gels have been used to treat the autoimmune chronic skin disease psoriasis [60,61,62]. The protective effect of SeNP against bleomycin-induced pulmonary fibrosis in the early and late stages has been studied [65]. In recent decades, the role of SeNP in neurological diseases has been actively studied, since neurons are especially vulnerable to damage caused by oxidative stress. Thus, this review provides ample evidence of the therapeutic properties of SeNP in the treatment of Alzheimer’s, Huntington’s, Parkinson’s diseases, epilepsy, and ischemia. This review also provides the latest data on the role of SeNP in the regulation of diabetes mellitus and various oncological diseases. There is a problem using Se in cancer therapy due to its toxicity and bioavailability, whereas unmodified and modified SeNP demonstrate antitumor activity against several cancer cell lines depending on time and dose. SeNP can significantly reduce the toxicity of elemental Se when used for chemotherapy.

## 5. Conclusions

In sum, the main purpose of this review was to systematize the latest data on the therapeutic effect of SeNP, a key trace element that plays an important role in human health. In addition, the most common and well-studied methods for obtaining these nanoparticles were presented. The data presented in the review will help researchers quickly navigate the field of existing research on the functions and therapeutic effects of SeNP and determine new directions for their research.

## Figures and Tables

**Table 2:** Physiological effects of SeNPs on neurodegeneration.

	Cellular Effects	Ref.
Alzheimer’s disease	↑ glutathione peroxidase ↓ ROS, [Ca^2+^]_i_, caspase-3, malondialdehyde, Zn^2+^-induced formation of Aβ40 fibrils, binding of Cu^2+^ to Aβ42 monomers, tau-hyperphosphorylation, Aβ1-42-induced cytotoxicity	[80,81,82,83]
Huntington’s disease	↑ antioxidant capacity, protective roles from behavioral dysfunction, the sensory and mechanical responses of ASH neurons ↓ ROS, neuronal death, alleviate behavioral dysfunction, aggregation of mutant huntingtin protein	[84],
Parkinson’s disease	↑ SOD, GSH-PX, Dopamine, ↓ ROS, MDA,	[85,86,87,88,89]
Epilepsy	↑ GPx, GSH-PX, COD2, NRF-2, HO-1, BCL-2, GABA, Ach, NE, 5-HT, ↓ ROS, NO, Hsp7, MDA, [Ca^2+^]_i_, IL-1β, TNFα, BAX, Glutamate, Caspase-3	[90,91,92,93]
Stroke	↑ CAT, SOD, GPx, GSH-PX, Se, ER-selenoproteins, BDNF ↓ [Ca^2+^]_i_, ROS, inflammation	[94,95,96,97,98,99,100,101,102,103,104]

**Table 3:** Physiological effects of SeNP in oncology.

	Cellular Effects	Ref.
Cancer	↑ ROS, NO, miR16, mitochondrial destruction, [Ca^2+^]_i_, CHOP, GADD34 BIM, PUMA, BAX, caspase-4, MAP3K5, MAPK-8, ATP-release ↓ cell proliferation, migration, invasion, CCND1, zinc-dependent metalloproteinases MMP2 and MMP9, cyclin-D1, Bcl-2	[147,151,152,153,154,155,156]

## Data Availability

The data presented in this study are available on request from the corresponding authors.
